# Overexpressed cold inducible RNA-binding protein improves cell viability and EGF expression in glial cells

**DOI:** 10.1186/s12860-022-00460-3

**Published:** 2022-12-16

**Authors:** Aijun Liu, Shouchun Li, Yongcheng Jiao, Haibo Kong, Zhiwen Zhang

**Affiliations:** 1 Department of Neurosurgery, the Chinese PLA General Hospital, No. 28, Fuxing Road, 100853 Haidian Beijing, China; 2 Department of Neurosurgery, Shanghai Fourth People’s Hospital Affiliated of Tong Ji University, Shanghai, China

**Keywords:** Cold-inducibleRNA-binding protein, neuroprotection, epidermal growth factor, cell viability

## Abstract

**Background:**

Cold inducible RNA-binding protein (CIRP) is a key protein in the hypothermic therapy. Highly expressed CIRP exerts a neuroprotective effect on neurons. The aim of this study is to provide the evidence of the protective effects of CIRP on the glial cells and explore the downstream pathway of CIRP.

**Results:**

The results of this study demonstrated that the cell viability of the glial cells with CIRP overexpression was increased significantly compared to the control. With CIRP overexpression, the epidermal growth factor (EGF) mRNA expression was found increasing significantly and the mRNA expressions of derived neurotrophic factor (BDNF), bcl-2, vascular endothelial growth factor (VEGF) and nerve growth factor (NGF) were not upregulated compared to the control. EGF and CIRP co-expression was demonstrated on the glial cells. With CIRP expression, EGF expression on the glial cells was increased statistically compared to the control.

**Conclusion:**

CIRP overexpression increases the cell viability of the glial cells, exerting a neuroprotective effect. EGF expression is activated on the glial cells with CIRP overexpression, implying a pathway of CIRP neuroprotection via EGF activation.

## Background

Cold inducible RNA-binding protein (CIRP) is an 18-kD protein consisting of an N-terminal RNA recognition motif and a C-terminal arginine-rich region. It’s primarily localized in the nucleus and can migrate to the cytoplasm binding mRNA. CIRP expression in the brain is increased significantly under cold stress [1, 2], and the upregulation of CIRP plays an important role on triggering the animal hibernation [3, 4]. These findings inspired the idea that highly expressed CIRP may exert neuroprotective function. Our previous studies demonstrated that CIRP can inhibit neuron apoptosis [5, 6], and Zhang et al [7] reported that overexpressed CIRP under hypoxia can restore the proliferation of neuronal stem cells suggesting a neuroprotective role of CIRP. Recently some studies indicated that extracellular CIRP can induce inflammatory responses resulting cells damage [8], then direct utility of CIRP as a neuroprotective factor seems unreasonable theoretically. We hypothesized that it was an ideal condition as a neuroprotective factor when CIRP was highly expressedintracellularly with minimal extracellular distribution. The pathway underlying intracellular CIRP expression may provide a new clue for a practical neuroprotection protocol. Our previous work only presented that the increased CIRP can reduce neuron apoptosis by increasing Bcl-2, Akt and pERK1/2 proteins [5]. More and more studies emphasized the role of glial cells on the neuroprotective function. In this study we investigated the effects of overexpressed CIRP on the cell viability of a glial cell line and the relationships between the CIRP expression and some neuroprotective factors, including epidermal growth factor (EGF), derived neurotrophic factor (BDNF), bcl-2, vascular endothelial growth factor (VEGF) and nerve growth factor (NGF).

## Materials and methods

### Cell culture

A glial cell line, SHG-44, was purchased from Shanghai GeneChem Co. Ltd. (Shanghai, China). The cells were cultured in 96-well plates at densities about 10,000 cells per well in GibcoDulbecco’s Modified Eagle Media (DMEM/F-12; Thermo Fisher Scientific) containing 10% fetal bovine serum (FBS, Thermo Fisher Scientific) supplemented with 1% penicillin/streptomycin (Thermo Fisher Scientific), at 37℃ in a humidified atmosphere with 5% CO_2_ in the air. The media was changed 2–3 times weekly and at 24 h before the cells were collected.

### Establishment of CIRP overexpression on SHG-44 cells

The recombinant lentiviral vector system containing the green fluorescent protein (GFP) reporter used in this study was purchased from Shanghai Genechem Co., Ltd. The vector encoding full length of CIRP gene (AB000362) was constructed, and PCR and DNA sequencing confirmed the accurate insertion of the CIRP cDNA. For transfection, the glial cells were plated in 96-well plates and transfected with either with CIRP lentiviral vectors (CIRP overexpression group, OE-CIRP) or with the GFP lentiviral vectors (negative GFP control group, N-GFP) in serum-free medium for 12 h at a multiplicity of infection (MOI) of 40. Polybrene (5ug/mL) was added to enhance the infection efficiency. The other cells without lentiviral infection served as a control group (control group, CON) and the cells transfected with naïve lentivirus served as a mock group (mock group, MOCK). The cells were then washed and embedded into complete medium. Four days after transfection, the GFP gene expression was examined using fluorescence microscopy. The transfection efficiency was calculated.

### Cell treatment

The OE-CIRP cells and MOCK cells were challenged with four injuries including oxidation, hypoxia, glucose deprivation and glutamate induced neurotoxicity. First, the MOCK cells were treated with various concentrations of H_2_O_2_, CoCl_2_ and glutamate respectively for 12 h to choose the suitable concentrations for further procedures. After the chosen concentrations confirmed, four injuries were applied. Oxidative stress was induced by H_2_O_2_ as following. The medium was removed firstly, then H_2_O_2_ was added on the cells. The medium was added immediately into the wells to get the final chosen concentration of H_2_O_2_. This procedure was repeated every 2 h until 12 h to further analysis. CoCl_2_ (232,696, Sigma-Aldrich) was prepared as aqueous solutions at chosen concentration to induce chemical hypoxia. The glial cells were treated with the CoCl_2_ solution for 12 h. The cells were cultured with glucose- and serum-free DMEM (11,966,025, Thermo Fisher Scientific) for 12 h to achieve glucose deprivation. The cells were exposed to glutamate at chosen concentration for 12 h to induce neurotoxicity.

### MTT assay for cell viability

Before performing MTT assay, the culture medium was replaced with 100 µL of fresh one. MTT solution (10 µL, 12 mM, Vybrant™ MTT Cell Proliferation Assay Kit, Thermo Fisher Scientific) was added to each well, including a negative control of 10 µL of the MTT solution added to 100 µL of medium alone. After incubating at 37 °C for 4 h, the cell viability was analyzed with a microplate reader (Fluoroskan Ascent, Thermo Fisher Scientific), absorbance at 570 nm.

### Real-time polymerase chain reaction

Total RNA was isolated, and cDNA was synthesized as we reported previously [1]. The primer sets used in this study were designed and provided by Sangon biotech, Shanghai China and listed in Table 1. Real-time reverse transcriptase (RT-PCR) analysis was performed using an SLAN Real-Time PCR System (Hongshi, Shanghai, China). PCR conditions used for amplification were as follows: initial denaturation at 95 °C for 5 min, 40 cycles of denaturation at 95 °C for 15 s, annealing at 65 °C for 30 s, and elongation at 84 °C for 30 s. Data were collected in real time at the end of each cycle. Negative controls, without reverse transcriptase, and water controls were included in each reaction. In addition to real-time and melting curve analysis of the reactions, amplified products were separated electrophoretically on 2% agarose gels with ethidium bromide and visualized under UV light to confirm proper amplicon size, as well as the absence of non-specific products. All PCR products produced a single specific product.

**Table 1:** Primer sets used in this study

No.	Primer	Sequences (5’to 3’)	Bases	Product size (bp)	
1	CIRP-f	GGAGACTCAACGATCCCGAG	20	318	
	CIRP-r	TGACTCCGGCTGGCATAGTA	20		
2	β-actin-f	GAAGTGTGACGTTGACATCCG	21	282	
	β-actin-r	GCCTAGAAGCATTTGCGGTG	20		
3	EGF-f	CATCGTGGTGGCTGTCTG	18	85	
	EGF-r	CTTCTGAGTCCTGTAGTAGTGG	22		
4	BDNF-f	GTGTGACAGTATTAGTGAGTGG	22	136	
	BDNF-r	CTTGGTCTCGTAGAAGTATTGC	22		
5	BCL-2-f	CTTGGTCTCGTAGAAGTATTGC	18	182	
	BCL-2-r	CTTCAGAGACAGCCAGGAG	19		
6	VEGF-f	GCTTACTCTCACCTGCTTCTG	20	89	
	VEGF-r	GGCTGCTTCTTCCAACAATG	19		
7	NGF-f	CCAAGGGAGCAGCTTTCTATC	20	110	
	NGF-r	GTGTGGTTCCGCCTGTATG	18		

Relative expression data were calculated according to the two-delta threshold (∆∆Ct) relative quantification method. Normalization of Ct values was performed by subtracting β-actin Ct from the CIRP Ct values acquired for each sample. To calculate the relative difference in the number of cycles between the samples, the average Ct value of the samples from the control group was selected as baseline. This number was subtracted from each of the Ct values that were previously normalized to β-actin. The relative difference between sample groups was calculated based on the difference in Ct values using 2-Δ(ΔCt).

### Double immunohistochemistry

The cells were incubated with PBS twice for 10 min each before being fixed with 4% paraformaldehyde at room temperature for 15 min and then washed with PBS containing 0.05% Tween20 to remove the fixative. The fixed cells were permeabilized with 0.1% Triton X-100 in TBS for 10 min at room temperature and blocked with 1% Blocker BSA for 15 min at room temperature. Anti-CIRP antibody (AB166775, Abcam) and anti-EGF antibody (701,538, Invitrogen) as primary antibodies were added to the respective wells and incubated overnight at 4 °C. After primary antibody incubation, the cells were washed with PBS, and then incubated with Alexa Fluor 488 donkey anti-goat IgG secondary antibody and Alexa Fluor Plus 594 Donkey anti-Rabbit IgG secondary antibody for 2 h at room temperature. The cells were washed with PBS before incubation for 1 min with DAPI nuclear stain (D9542, Merck). Images were taken on an Olympus confocal microscope.

### Western blot

The cells was washed twice with cold PBS (0.01 M pH7.2～7.3), resuspended in ice-cold cell lysis buffer (9803, Cell Signaling Technology) with 1mM PMSF (8553, Cell Signaling Technology) and incubated at 4 °C for 30 min. The lysates were centrifuged at 12,000 rpm for 10 min at 4 °C. The protein of the supernatants was stored at − 80 °C. CIRP and EGF levels were analyzed by Western blotting with monoclonal antibody to CIRP ((AB166775, Abcam) and EGF (701,538, Invitrogen). β-Actin was used as the internal control. Blot bands were quantified using an optical scanner and the Labworks system (Labworks Inc, Winnipeg, Manitoba, Canada).

#### Statistical analysis

Statistical analysis was performed using Sigma Plot (12.5) for Windows. Data were presented as the mean ± SD of three independent experiments. Statistical analysis of differences was carried out by a one-way ANOVA. *P* < 0.05 was considered to indicate statistical significance.

## Results

### Cell transfection and overexpression of CIRP on the glial cells

**Fig. 1:**
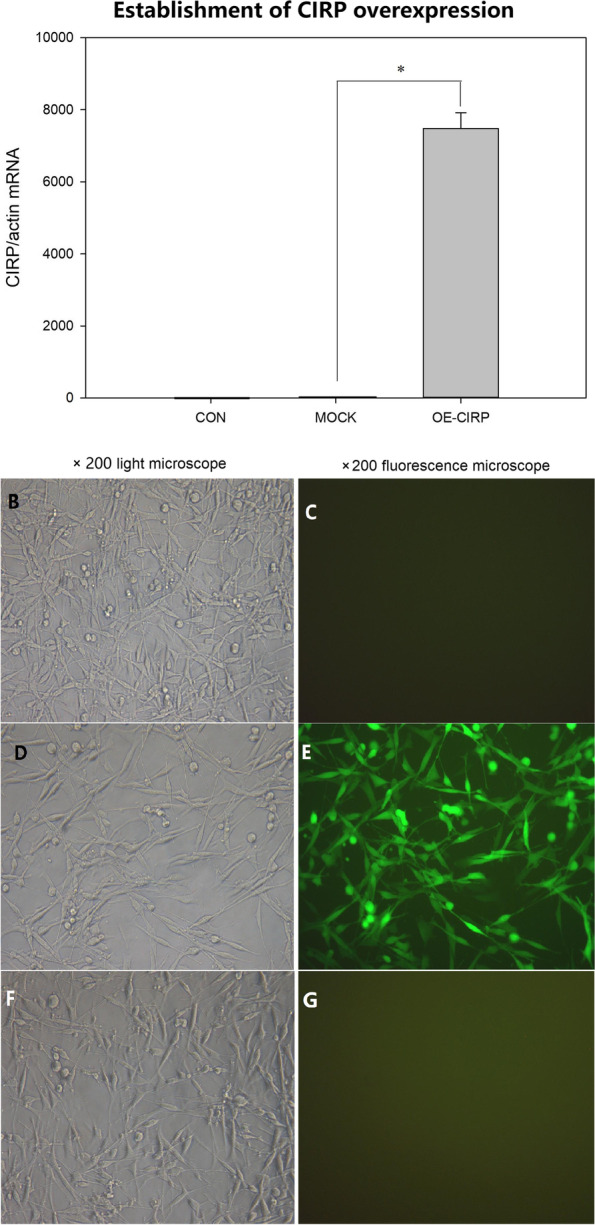
Overexpression of CIRP mRNA in glial cell line SHG-44. **A** Comparison of CIRP mRNA expression between the normal control cells (CON)(*n* = 3), the negative control cells transfected with green fluorescence protein (N-GFP) (*n* = 3) and the cells transfected with CIRP (OE-CIRP) (*n* = 3) *P < 0.01. **B** Normal SHG-44 cells under light microscope (×200). (C) Normal SHG-44 cells under the fluorescence microscope (×200). **D** SHG-44 cells with GFP transfection under light microscope (×200). **E** SHG-44 cells with GFP transfection under fluorescence microscope (×200). **F** SHG-44 cells with CIRP transfection under light microscope (×200). **G** SHG-44 cells with CIRP transfection under fluorescence microscope (×200)

The cells in culture presented the properties of the glial cells under light microscope (Fig. 1B, D, F). After 4 days transfection with GFP or CIRP lentiviral vectors, the cells with GFP expression was observed under the fluorescence microscope (Fig. 1 C, E, G). The efficiency of the infection was above 80% at a MOI of 40. RT-PCR analysis demonstrated that the relative levels of CIRP mRNA were markedly increased in CIRP group (5476.56 ± 442.8) compared with the levels in the GFP control group (1.07 ± 0.12) (Fig. 1 A).

### Cell viability of the glial cells with CIRP overexpression

**Fig. 2:**
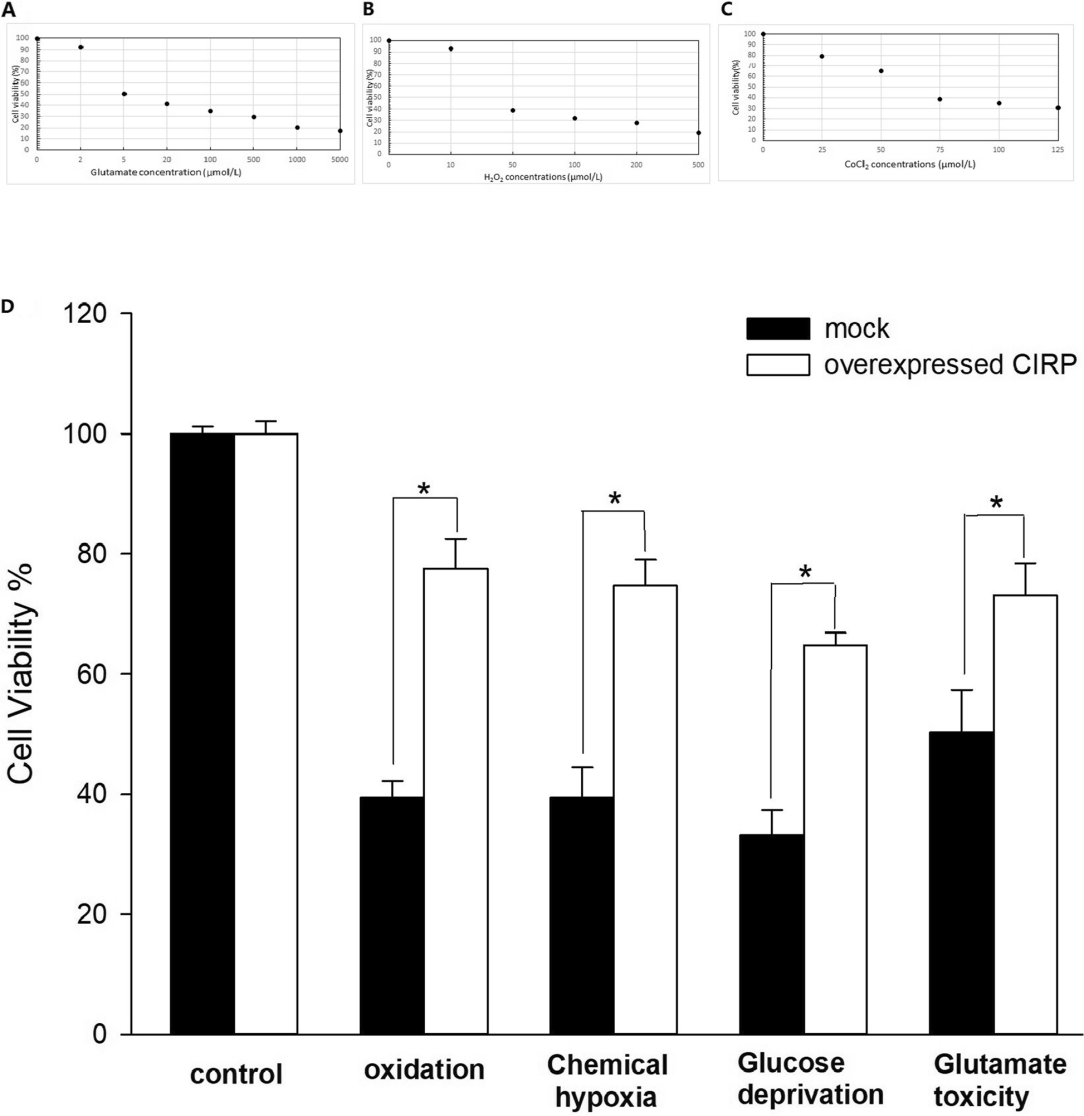
The effect of various concentrations of H_2_O_2 _(**A**), CoCl_2 _(**B**) and glutamate (**C**) on glial cells. Cell viability of the glial cells after the treatment of oxidation, hypoxia, glucose deprivation and glutamate toxicity (**D**). Experiments were repeated three times. Bars indicated means ± SD. *p<0.05

Cell viability of the MOCK cells was reduced as the concentrations of H_2_O_2_, CoCl_2_ and glutamate increased (Fig. 2 A-C). The concentrations for around 50% cell viability were chosen as the suitable concentrations. After exposure of the glial cells to the oxidation produced by 50µmol/L H_2_O_2_for 12 h, the overexpressed CIRP increased the cell viability significantly compared to the control (77.6 ± 4.9% versus 39.4 ± 2.8%)(Fig. 2D). After 12 h chemical hypoxia induced by 75µmol/L CoCl_2_, the overexpressed CIRP also increased the cell viability of the glial cells significantly compared to that of the control cells (74.7 ± 4.3% versus 39.4 ± 5.1%)(Fig. 2D). After 12 h glucose deprivation treatment, the overexpressed CIRP increased the cell viability significantly compared to the control (64.8 ± 2.1% versus 33.2 ± 4.2%)(Fig. 2D). And the overexpressed CIRP increased the cell viability statistically compared to the control cells (73.2 ± 5.2% versus 50.3 ± 7.1%) after the treatment of 5µmol/L glutamate for 12 h(Fig. 2D).

## Expression of growth factors and bcl-2 after CIRP overexpressed on the glial cells

**Fig. 3:**
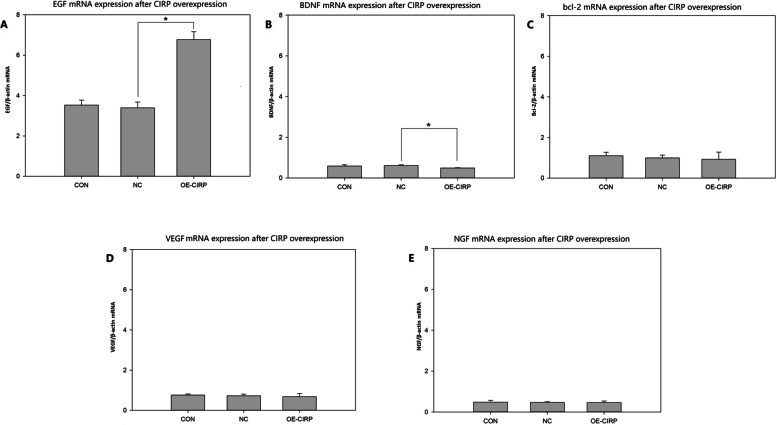
RT-PCR analysis for the expression of some neurotrophines and cytokines after CIRP overexpressed on the glial cell line SHG-44. **A** EGF mRNA expression(*n* = 3), *p < 0.05. **B** BDNF mRNA expression (n = 3), *p < 0.05. **C** bcl-2 mRNA expression (n = 3). **D** VEGF mRNA expression (*n* = 3). **F** NGF mRNA expression (*n* = 3) CON: normal control cell group; NC: negative control cell group, cells transfected with negative control lentiviral vectors; OE-CIRP: overexpressed CIRP cell group, cells transfected with CIRP lentiviral vectors

The expression of several growth factors and bcl-2, which have neuroprotective effect, were analyzed on the CIRP overexpressed glial cells. The relative mRNA expression of EGF on the cells with CIRP overexpression was increased significantly compared to the negative control (Fig. 3 A). The relative mRNA expression of BDNF on the cells with CIRP overexpression was decreased statistically compared to the negative control (Fig. 3B). And there was no statistical difference in the relative mRNA expression between bcl-2(Fig. 3 C), VEGF(Fig. 3D), NGF(Fig. 3E) and their negative control group.

## CIRP overexpression increased EGF expression on the glial cells

**Fig. 4:**
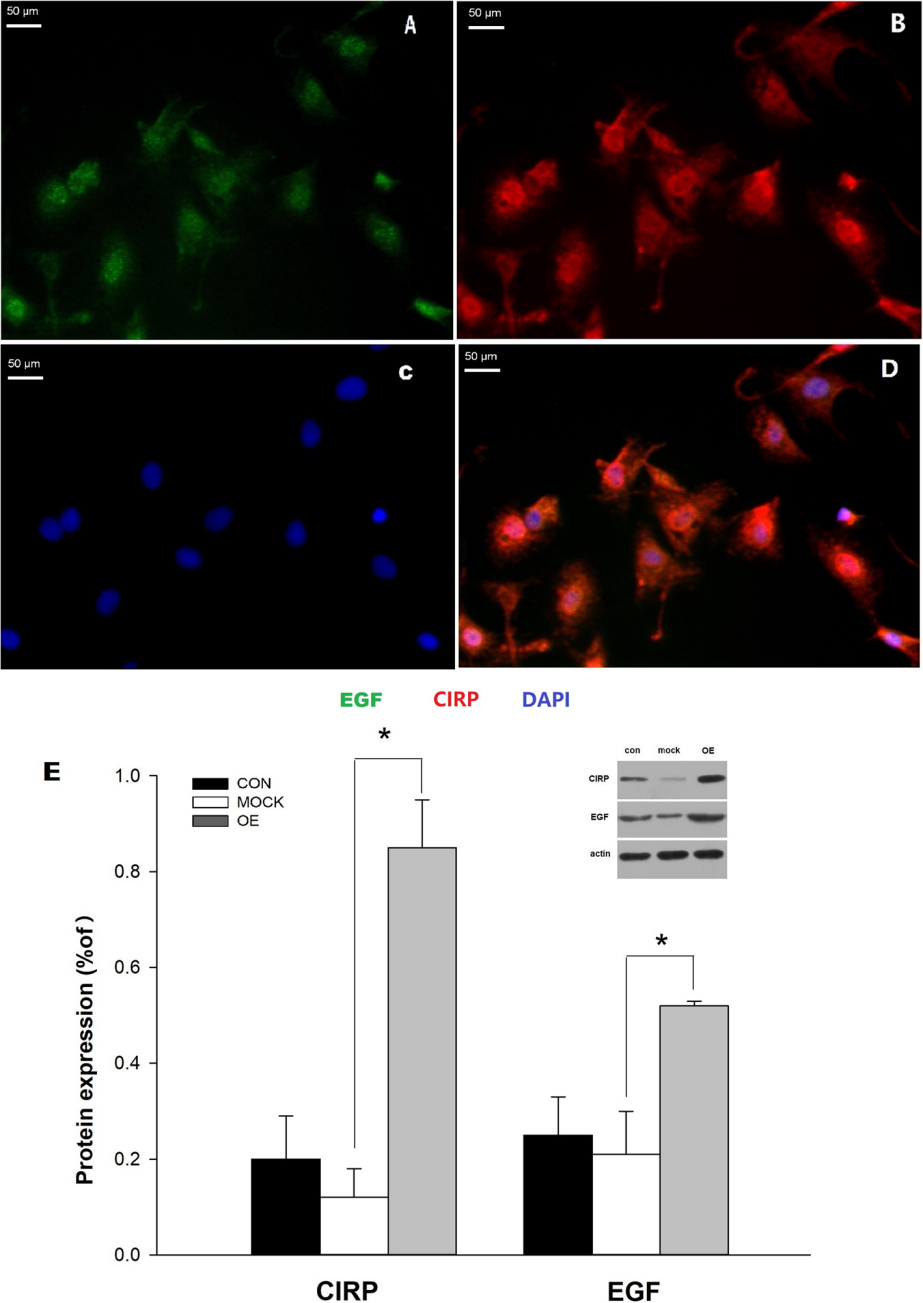
CIRP overexpression increased EGF level on the glial cells. Double-labeling immunofluorescence staining and confocal microscopy to EGF (green **A**), CIRP (red **B**), DAPI staining (blue **C**)and merge (**D**). Western blot analysis of CIRP and EGF, and the relative expression of CIRP and EGF **E**. CON: normal control group, MOCK: negative control group, OE: overexpression of CIRP group. Experiments were repeated three times. Bars indicated means ± SD. *p<0.01

After 4 days transfection with CIRP lentiviral vectors, the glial cells were performed double immunohistochemistry for CIRP and EGF. The double staining demonstrated that CIRP was expressed strongly on the glial cells (Fig. 4B), and simultaneously, EGF was also expressed significantly on the glial cells (Fig. 4 A). The distribution of CIRP and EGF on the glial cells almost overlapped in the merged image, CIRP dominantly in the nucleus and dotted EGF around the nucleus(Fig. 4D).Western blot analysis on these cells showed that the CIRP expression in the overexpression group was increased significantly compared to the negative control group (0.85 ± 0.1% versus 0.12 ± 0.06%) (Fig. 4E), and Simultaneously the EGF expression in the CIRP overexpression group was also increased significantly compared to the negative control group (0.52 ± 0.01% versus 0.21 ± 0.09%) (Fig. 4E).

## Discussion

Most of the preclinical studies demonstrated that induced therapeutic hypothermia exerted neuroprotective effects on different brain injuries. Therapeutic hypothermia is usually recommended as a treatment for head injury and cerebral ischemic stroke in clinical practice. However, the updated guidelines for the treatment of severe traumatic head injury [9] or acute ischemic stroke [10] only list therapeutic hypothermia as a level BII recommendation which means a low-quality body of evidence. The limitations of therapeutic hypothermia when being applied in the clinical practice are mainly attributed to body shivering, consciousness alteration and other complications when body temperature decreasing. The concept of “targeted temperature management” [11, 12] was emerging in recent years trying to overcome the limitations [13–15], but the outcomes still cannot catch up with the demands of clinical practice. Precise control of the brain temperature seems the best way to achieve the neuroprotective effect while avoiding the complications due to low body temperature. CIRP now is considered as a possible promoter for cold stress and an ideal manipulating target for “targeted temperature management”. But the biological activation protocol and downstream pathways of CIRP still need to be elucidated.

At molecular level, CIRP is a potential target for therapeutic manipulation to achieve the effect of low brain temperature. Due to extracellular CIRP could induce severe inflammatory response and damage the cells [8], the safety of highly expressed CIRP on cells should be verified at first. Our research demonstrated that overexpressed CIRP intracellularly didn’t affect the glial cell growth morphologically (Fig. 1). Moreover, the glial cells with overexpressed CIRP presented increased viability under different harmful conditions such as oxidation, hypoxia, glucose deprivation and glutamate toxicity (Fig. 2). Our previous works had proved that CIRP could protect the cerebral neurons from H_2_O_2_ induced apoptosis [5, 6]. The results of this study provided the direct evidence that CIRP had a significant neuroprotective effect on the glial cells. The role of glial cells in the central nervous system (CNS) has attracted more and more attention in recent years. The glial cells in the brain are considered as an important component to modulate the functions of neurons besides sustaining the neuronal networks and homeostasis [16–18]. Toriuchi K. et al [19] demonstrated that under hypothermic conditions, the astrocytes with increased erythropoietin expression exerted a neuroprotective effect on the neurons. Emerging studies considered the glial cells as attractive targets for therapeutic drugs in brain disorders and injuries [18]. The present study supported the idea that manipulating the CIRP of glial cells was a potential path to protect the brain function after different injuries. Intracellular CIRP overexpression prolongs G1 phase of the cell cycle and suppresses the cell growth rate, which could decrease the energy demand of the cells [20]. This mechanism could partly explain the increased cell viability of the glial cells with CIRP overexpression.

At present, it is widely accepted that cold stress, ultraviolet radiation, and some extent of hypoxia could induce the CIRP expression on different cells [21]. But the exact mechanisms of how these physical stresses work on the CIRP expression are still unknown and the effects of manipulated CIRP overexpression on the cells are also needed to be elucidated. Previous studies [22] demonstrated that CIRP is one of the limited genes whose expressions increased during cold exposure. CIRP might be a promoter to activate downstream pathways to accommodate hypothermia. We have investigated CIRP since 2006, but we did not find any effective ways to biologically activate CIRP expression besides lentivirus transfection. The downstream pathway of CIRP is also a rational manipulating point to achieve therapeutic hypothermia. To begin with, we wanted to know if CIRP could trigger any well-known neuroprotective factors and cytokines specifically to exert its function. We analysed the gene expression of EGF, VEGF, NGF, BDNF and bcl-2 on the glial cells with CIRP overexpression. The results demonstrated that only EGF mRNA expression and EGF expression were increased significantly (Figs. 3 and 4). EGF is a widely investigated factor playing a key role in promoting cell proliferation and opposing apoptosis by activation of EGFR family. EGF acts on several mature neurons of the CNS in a neurotrophic manner, increasing the survival and process outgrowth of the neurons and preventing the death of the neurons from neurotoxicity, oxidation and other harmful conditions [23]. Interestingly, the neuroprotective effects of EGF on the neurons result not only from direct action on the neurons but also from a cell-to-cell effect through glial cells [23]. Under the circumstances of intracellular CIRP overexpression at the normal temperature in this study, EGF mRNA and EGF expression were increased significantly, and the distribution of CIRP and EGF in the glial cells almost overlapped (Fig. 4D). Some literatures reported that during cold stress, only the expressions of CIRP, RBM3 and NF-1 variant were found increased in cells [22]. Then we hypothesized that in the time of rewarming, activated CIRP migrated in the cytoplasm, and could trigger EGF expression immediately. This pathway might play an important role for protective effects on the glial cells and neurons. Kaija H. et al. [24] reported that Relative mRNA expressions of amphiregulin (AMR), cyclin D1 (CyD1), cyclin-dependent kinase inhibitor 1 A (p21), transmembrane form of the prostatic acid phosphatase (PAcP), thrombomodulin (TM) ,heat shock transcription factor 1 (HSF1) and Bax/Bcl-2 were increased significantly after a hypothermia-rewarming process in healthy rat prostate tissue. It is still uncertain how such hypothermia-rewarming process, mimicking clinical hypothermic therapy, might affect the gene expressions of the neurons and glial cells. EGF now is an indispensable reagent in the field of cell culture to enhance the differentiation, maturation and survival. But single EGF is unable to accomplish this complex mission without the coordination of other cytokines and factors. Further studies are required to completely understand the downstream pathways of CIRP activation.

## Conclusion

The present study demonstrated that through transfection with CIRP lentiviral vectors, CIRP mRNA expression was increased significantly in the glial cells. The overexpressed CIRP in the glial cells increased the cell viability under oxidation, chemical hypoxia, glucose deprivation and glutamate neurotoxicity, while CIRP overexpression also increased the EGF mRNA and EGF expression in the glial cells, implying that EGF may play a key role as a neuroprotector after CIRP activation.

## Data Availability

The data that support the findings of this study are available from the corresponding author AL, upon reasonable request.
